# Research progress of CD80 in the development of immunotherapy drugs

**DOI:** 10.3389/fimmu.2024.1496992

**Published:** 2024-11-07

**Authors:** Lanying Li, Lei Yang, DePeng Jiang

**Affiliations:** Department of Respiratory Medicine, The Second Affiliated Hospital of Chongqing Medical University, Chongqing, China

**Keywords:** CD80, immunotherapy drugs, T cells, tumor, autoimmune diseases

## Abstract

CD80 is a molecule that plays an important role in the immune system, especially during T-cell activation, and its ligands are mainly CD28, PD-L1, and CTLA-4. CD80 is expressed on the surface of tumor cells, and it can be used as a molecular target in the process of T-cell anti-tumor immune response. In autoimmune diseases, CD80 can also regulate autoimmune diseases by modulating immunity. This review mainly focus on the role of CD80 in the immune system, as well as the research progress on the application of CD80-related immunopharmaceuticals in the treatment of tumors and autoimmune diseases.

## 1 The role of CD80 in the immune system

CD80, also known as B7, B7.1, or BB1, is a member of the immunoglobulin superfamily with a size of 44-54 kDa and is expressed by a variety of cells, including activated B cells, T cells, macrophages, dendritic cells, and tumor cells (1–3). Its receptors are mainly CD28, PD-L1, and CTLA-4 (4–6). CD80 plays a crucial role in T cell activation.

### 1.1 CD80 interacts with CD28 molecules on the surface of T cells

The molecule now known as CD80 was initially described as a human B cell-associated activation antigen (7). Previous studies have shown that the CD80 molecule on the surface of B cells acts as the receptor for the CD28 molecule on the surface of T cells. Blocking the interaction of CD28 with its natural ligand CD80 has been found to weaken the activity of human T lymphocytes (8, 9). T-cell activation requires the activation of dual signaling systems. The TCR-CD3 complex combined with the antigen peptide-MHC complex mediates the first signal for T cell activation. The second signal involves the combination of multiple pairs of costimulatory molecules on T cells and antigen-presenting cells, with the most important of which is the combination of the CD28 costimulatory molecule on the surface of T cells and the CD80 molecule on the antigen-presenting cells (10). In the dual signaling system of T-cell activation, the absence of CD28 activation results in excessive activation-induced cell death (AICD). However, after CD80 binds to CD28, the AICD of T cells can be avoided, leading to more durable anti-tumor activity of T cells (11). Furthermore, the combination of CD80 and CD28 can also enhance the secretion of cytokines such as IL-2 by T cells. Moreover, it can enhance the proliferation of CD4+ T cells and the cytotoxic activity of both CD4+ and CD8+ T cells (4). Recent studies have shown that inadequate activity of the costimulatory molecule CD28 on T cells can lead to a decrease in the anti-tumor activity of T cells (12). However, following an increase in CD28 activation signal, the anti-tumor activity of T cells is enhanced (13, 14). Consequently, the activation of the CD28 molecule on the surface of T cells by CD80 may enhance the killing efficiency of T cells against solid tumors, offering a new approach to immunotherapy.

### 1.2 CD80 interacts with PD-L1 on the surface of tumor cells

Programmed cell death 1 ligand 1 (PD-L1) is also known as cluster of differentiation 274 (CD274) or B7 homolog 1 (B7-H1). It is a protein encoded by the CD274 gene in humans. As early as 1999, researchers first defined a previously unknown costimulatory molecule, PD-L1, as the third member of the B7 family. They suggested that B7-H1 (PD-L1) may be involved in the negative regulation of cell-mediated immune responses (15). Subsequently, some researchers have found that the combination of PD-L1 with its ligand PD-1 can lead to the suppression of T cell receptor-mediated lymphocyte proliferation and cytokine secretion. In addition, PD-1 signaling can partially inhibit the level of CD28-mediated costimulation (16). The interaction between PD-L1 on tumor cells and PD-1 on the surface of T cells can result in T cell immunosuppression. The mechanism involves the interaction between PD-1 on the surface of T cells and PD-L1 on the surface of APC cells, which leads to PD-1 tyrosine phosphorylation and recruitment of SHP2. This process inhibits the activation of TCR and CD28, thereby reducing T cell immune response (17, 18). The failure of T cells’ immune response contributes to tumor metastasis, invasion, and recurrence during the anti-tumor immune process.

However, Haile ST et al. discovered that by transfecting human tumor cells with a gene encoding the costimulatory molecule CD80, they could reduce PD-L1-mediated immunosuppression by tumor cells and restore T-cell activation (19). Subsequently, Chaudhri A et al. found that PD-L1-CD80 could interact in cis within the same cell, but not in trans between two cells. This competitive interaction blocks the binding of PD-L1 to PD-1 or B7-1 to CD28 (6). Recent studies have shown that the interaction between CD80 and PD-L1 interferes with the binding of PD-L1 of tumor cells to the inhibitory receptor PD-1 on T cells, thereby promoting the immune response of T cells (20). By expressing CD80 and PD-L1 on the surface of the same APC cells *in vitro*, some researchers have found that they bind in a cis-acting manner to form a CD80-PD-L1 cis-heterodimer, which plays an important role in the experiment. On one hand, the CD80 cis-heterodimer can activate the costimulatory molecule CD28 and maintain the function of T cell activation. On one hand, the CD80 cis-heterodimer can activate the costimulatory molecule CD28 and maintain the function of T cell activation. On the other hand, the interaction between CD80 and PD-L1 interferes with the binding of PD-L1 on tumor cells to the inhibitory receptor PD-1 on T cells, thereby promoting T cell immune response (20, 21). Additionally, it can also inhibit the T cell immunosuppressive CTLA-4 pathway (21). In addition, the results were validated in a mouse model. It was found that introducing mutations in the interaction site on PD-L1 or on CD80 significantly suppressed the anti-tumor immune response in mice. In other studies, it has also been reported that PD-L1 on T cells interacts in trans with CD80 on APCs, and blocking this trans interaction enhances anti-tumor immunity (22). As early as 2018, researchers combined CAR-T with PD-1-blocking scFv to treat tumors and achieved significant anti-tumor efficacy (23). The role played by CD80 is similar to that of PD-1-blocking scFv, and we guess that blocking the interaction between PD-L1 and PD-1 by CD80 may have better anti-tumor efficacy than PD-1-blocking scFv. By applying CD80 antibodies, fusion proteins, or combining with CAR-T or other therapies, CD80 may offer a new direction for tumor immunotherapy.

### 1.3 CD80 interacts with CTLA-4 on the surface of T cells

Linsley, P et al. discovered that CD80 can bind not only to PD-L1 on the surface of T cells but also interact with the inhibitory molecule CTLA-4 on the surface of T cells (5). It has also been demonstrated that the level of T cell antigen receptor stimulation is regulated by the CD28 costimulation signal and the CTLA-4 inhibitory signal. Compared to the T cell surface molecule CD28, its inhibitory surface molecule CTLA-4 has a stronger binding affinity to CD80 (24, 25). Some scholars have reported the crystal structure of the human CTLA-4/CD80 costimulatory complex at a resolution of 3.0 A. They found that CTLA-4 and CD80 are arranged in a surprising periodic pattern in the crystal lattice, where a bivalent CTLA-4 homodimer bridges a bivalent CD80 homodimer (26). Further studies have shown that CTLA-4 indirectly inhibits CD28 signaling by blocking the APC surface molecule CD80 through endocytosis (27, 28). This negative regulation was also observed by other researchers, who found that CTLA-4 inhibited numerous T cell-dependent immune responses *in vitro* and *in vivo* after binding to CD80 with high affinity (29, 30). Recent studies have found that PD-L1: CD80 cis-heterodimerization inhibits PD-L1: PD-1 and CD80: CTLA-4 interactions through different mechanisms, but still allows CD80 to activate T cell costimulatory receptors (21). The increased interaction among CD80, PD-L1, and CTLA-4 presents a new concept for anti-tumor immunotherapy.

### 1.4 CD80 as a target

#### 1.4.1 CD80 as a tumor target

CD80 is expressed in a variety of blood tumors and solid tumors, such as Hodgkin lymphoma, non-Hodgkin lymphoma, pancreatic cancer, breast cancer, lung cancer, etc (31–36). Targeting CD80, or triggering natural killer cell-mediated killing of cancer cells by inducing CD80 expression, has been explored as a possible cancer immunotherapy.

CD80 is expressed in hematoma cells. Dakappagari N et al. found that CD80 was expressed in both malignant B cells and non-malignant stromal cells of non-Hodgkin lymphoma (33). CD80 expression was also found in Reed-Sternberg(R-S) and immune cells in 50 histopathologically confirmed cases of Hodgkin’s lymphoma (31). Some researchers have found that the IL-4/anti-CD40 stromal cell culture system can induce high expression of CD80 in low-grade B-cell lymphoma under *in vitro* culture conditions, indicating that it is a potential immunotherapeutic target (37). Vooijs WC et al. constructed an anti-B7-1 immunotoxin containing an anti-CD80 monoclonal antibody and saponin as a toxin component, which has a similar affinity to natural MAb. It can show strong cytotoxicity to CD80+ B cell line Raji cells, Reed-Sternberg(R-S) cells, and CD80-transfected epithelial cell line A431 (32). These findings suggest that CD80 may be a potential target for the treatment of hematological tumors, especially B-cell lymphomas.

CD80 also plays an important role in solid tumors. It has been found that low expression of CD80 in tumor stem cells may inhibit the activation of the CD28 molecule on T cells in glioblastoma (38). Data have shown that the CD80 of pancreatic cancer cells is upregulated after treatment with TGF-β, and this is required for migration and invasion of pancreatic tumor cells *in vitro (* 34). The high expression of CD80 in cutaneous squamous cell carcinoma cells leads to the weakened killing of T cells against tumor cells by contact with CTLA4 (39). The researchers also shed light on the relationship of CD80 with CD28 and CTLA4. When CTLA4-mediated interactions with squamous cell carcinoma cells were blocked *in vitro*, tumor-CD80 engaged instead with CD28 on activated T cells. Both CTLA4 and CD28 are expressed by T cells. CD80 activates T cells via CD28 and inhibits T cells via CTLA4. However, CD80’s affinity is higher for CTLA4 than for CD28 (25). In addition, T cells display markedly elevated CTLA4 when contacted with tumor cells (40). Thus, within the tumor, the CD80 is more likely to engage CTLA4 than CD28, thereby dampening the function of T cells at attacking the tumor. To investigate the function of CD80 in lung adenocarcinoma, Feng W et al. collected transcriptome data along with corresponding clinical information from 594 lung samples in the Cancer Genome Atlas (TCGA) database. By using bioinformatics methods, elevated CD80 was found to improve the prognosis of patients with lung adenocarcinoma. It suggests that CD80 may be a potential prognostic and therapeutic target in lung adenocarcinoma (36). Similarly, some researchers analyzed the transcriptome profiles and related clinical information of 1090 breast cancer patients recorded in the Cancer Genome Atlas database and found that the expression of CD80 was closely related to the malignant degree of breast cancer, which also indicated that CD80 may be a promising target for immunotherapy strategy (35). In this study, they found that CD80 expression was elevated in basal-like and HER2-enriched subtype when compared with the luminal A subtype. Furthermore, CD80 showed higher expression in triple-negative breast cancer (TNBC) when compared with the non-TNBC group. In addition, they also observed elevated expression of CD80 in higher tumor grades. The results indicated a positive correlation between the expression of CD80 and the degree of tumor malignancy. Some researchers also studied 119 patients with primary soft tissue tumors and found that the high sCD80 (soluble form of CD80) group had significantly lower metastasis-free survival (MS) and overall survival (OS) at 5 years than the low sCD80 group (41). Interestingly, cells of different tumor types seem to evade antitumor immunity via disparate expression of CD80. Overexpression of CD80 on different types of tumors is not always negatively associated with patient prognosis. For example, the high expression of CD80 on the cell surface of lung adenocarcinoma significantly improved the overall survival of patients, while in breast cancer and squamous cell carcinoma of the skin, CD80 overexpression was an indicator of poor prognosis. Therefore, different types of tumors are closely related to the selection of our subsequent targeting strategy. For tumors where high CD80 expression is associated with a poor prognosis, we can directly choose CD80 as the targets. Conversely, for tumors where high CD80 expression can improve patient prognosis, we guess that they may benefit from immune checkpoint inhibitors, such as antibodies targeting CTLA4 or PD-1/PD-L1. The role of CD80 in solid tumors also suggests that it may be a promising target for solid tumor immunotherapy strategies.

#### 1.4.2 CD80 as a target in autoimmune diseases

In addition, the upregulation of CD80 is associated with a variety of autoimmune diseases, including multiple sclerosis, systemic lupus erythematosus, glomerular diseases, etc. The reason may be that in these diseases, T cells are overactive, and CD80 is closely related to T cell activation (42–44). CD80 has also been shown to contribute to the transmission of HIV infection *in vivo (* 45). The complex role of CD80 in the regulation of the immune system provides an important target for the treatment of various diseases.

The number of CD80+ lymphocytes in the blood increased significantly during the exacerbation of multiple sclerosis, and after IFN-β treatment, the number of CD80+ lymphocytes in the blood decreased significantly, which initially indicated that the number of CD80(+) cells may be an indicator of whether IFN-β treatment is effective (46). Subsequently, some studies have found that CD80+ B cells can be used as a possible therapeutic target for HTLV-1-related myelopathy/tropical spastic paraparesis and multiple sclerosis (43). CD80 is also expressed on antigen-presenting cells (APCs) of patients with Minimal Change Nephropathy. Various glomerular disease models associated with proteinuria have shown that increased urinary CD80 is closely related to patients with frequent recurrence of Minimal Change Nephropathy, and the current efficacy of CD80 inhibitors (abatacept) encourages further research on CD80 as a therapeutic target for patients with Minimal Change Nephropathy (42). The abnormality of T cell costimulatory molecules plays an important role in the immune pathogenesis of SLE. Studies have found that the expression of CD80 on the surface of T cells in patients with systemic lupus erythematosus is increased, and CD80 is mainly expressed on CD4+ T cells. Its increased expression is related to the disease activity of SLE (44, 47–49). The abnormal expression of CD80 in autoimmune diseases makes CD80 a potential and effective target for autoimmune diseases.

## 2 Immunotherapy drugs related to CD80

### 2.1 fusion protein

#### 2.1.1 Application of CD80 fusion protein in tumor treatment

As early as 2001, researchers utilized CD80 immunoglobulin G fusion protein to treat myeloid leukemia. They discovered that it could enhance the costimulatory activity of T cells and reinstate the expression of the costimulatory molecule CD80 on human AML blasts (50). Subsequently, researchers constructed a soluble protein consisting of the extracellular domain of human or mouse CD80 fused to the Fc domain of IgG1. This protein binds to PD-L1, inhibiting the interaction between PD-L1 and PD1, and promoting T cell activation through CD28 costimulation. Moreover, compared to the treatment with PD-1 or PD-L1 monoclonal antibodies, CD80-Fc is more effective in preventing PD-1/PD-L1-mediated inhibition and restoring T-cell activation (51). The team also demonstrated that soluble CD80 has a therapeutic effect in murine tumors *in vivo*. This effect was attributed to its ability to inhibit PD-1-mediated suppression and simultaneously activate CD28-mediated activation. This was achieved through the activation of downstream signaling pathways, such as transcription factors EGR1-4, NF-κB, and MAPK, which are involved in T-cell activation. Furthermore, soluble CD80 did not inhibit T cell function by interacting with CTLA-4, suggesting that CTLA-4 acts as a decoy receptor for CD80 rather than as an inhibitory signaling receptor (52).

The positive outcomes of prior preclinical studies have rapidly promoted CD80-related fusion proteins into clinical research. In 2019, initial data of FPT155 (NCT04074759), a phase I trial of a CD80 fusion protein, were released. The trial focused on its results in patients with advanced solid tumors. FPT-155 is a native CD80 fusion protein that functions by (i) enhancing its costimulatory T cell activity without inducing T cell hyperactivation through binding to CD28, and (ii) preventing CTLA-4 from competitively binding to endogenous CD80, thereby allowing CD28 signaling to become dominant in T cell activation in the tumor microenvironment. FPT155 not only showed long-lasting antitumor activity but was also well tolerated, with no dose-limiting toxicity or signs of clinical or laboratory cytokine release syndrome. In 2021, a new clinical study of the CD80 fusion protein ALPN-202 (NCT04186637) for the treatment of patients with advanced solid tumors published phase 1 clinical trial data. The data showed that the disease control reached 60%, and the trial found no safety concerns related to cytokine risk. Unlike FPT155, ALPN-202 is a mutated CD80-Fc fusion protein designed to overcome checkpoint inhibitor resistance by enhancing CD28 costimulation in the tumor microenvironment while inhibiting PD-L1 and CTLA-4. Peripheral immunoassays demonstrated evidence of CD28 activation and other associated immune activation, including elevated ICOS and Ki-67, as well as increased TCM and decreased Treg. The data indicates that in the treatment of solid tumors, PR accounts for 4%, SD accounts for 57%, and PD accounts for 39%. In addition, GI-101 (NCT04977453), a native CD80-Fc fusion protein, is currently in phase 1/2 clinical trials. The study aims to evaluate the safety, tolerability, pharmacokinetics, and therapeutic activity of GI-101 as a single agent or in combination with an anti-PD-1 antibody, tyrosine kinase (RTK) inhibitor, or local radiotherapy (RT) in a range of advanced and/or metastatic solid tumors. It is currently under recruitment. GI-101 is a CD80/IL2 fusion protein, which contains the Fc segment of IgG4. The introduction of IgG4 Fc makes the fusion protein safer and does not cause ADCC. On the other hand, it also enhances the half-life of the fusion protein so that it does not break down quickly in the body. In conclusion, the potential risk and side effect of the fusion protein is the over-activation of T-cells caused by CD28 activation, which leads to cytokine storm, but this situation is controllable by dose adjustment, and the benefit is much greater than the risk. In addition, the fusion protein has a half-life *in vivo*, and will not produce long-term impacts.

Although numerous immune checkpoint inhibitors, such as CTLA-4, PD-L1, and PD-1 inhibitors, have been introduced to the market, the majority of tumor patients will experience tumor recurrence, metastasis, and drug resistance. This may be attributed to the inadequate activation of T cells in the tumor microenvironment, such as insufficient CD28 costimulation signal, or the presence of immunosuppressive T cells in the immunosuppressive microenvironment, characterized by elevated levels of LAG3, PD-1, and TIM3. The combination of CD80 fusion protein with CTLA-4/PD-1/PD-L1 inhibitors, the development of bi- or tri-specific antibodies, or the combination of targeted drugs has the potential to overcome the poor efficacy of PD-1 inhibitors alone.

#### 2.1.2 Application of CD80-associated fusion proteins in the treatment of autoimmune diseases

Fusion proteins associated with CD80 can also be utilized in the treatment of autoimmune diseases, including rheumatoid arthritis (RA), renal transplant rejection, psoriatic arthritis (PsA), and others (53–55). Currently, only Belatacept from Bristol-Myers Squibb is available on the global market. Belatacept is a soluble fusion protein that contains the CTLA-4 receptor on the surface of T cells in the upper part of its structure. This protein binds to CD80 on the surface of APC and inhibits the activation of CD28 on the surface of T cells. So that T cells cannot be activated to function. The lower half of the belatace comprises the Fc portion of the IgG1 antibody, which is utilized to extend the half-life and enhance the stability of the drug. Belatacept, an enhanced version of abatacept, differs from it by only 2 amino acids. Its inhibition of T cell activity is 10 times greater than that of abatacept, and its inhibition of CD80 is 2 times greater than that of abatacept (56). In 2020, Belatacept received clinical approval from the China National Medical Products Administration for the prevention and treatment of autoimmune indications, including renal transplant rejection and rheumatoid arthritis. They also plan to study the clinical use of belatacept to reduce the side effects of tumor immunity. In 2021, a phase II trial of abatacept showed that it could significantly improve transplantation outcomes associated with acute graft-versus-host disease (AGVHD) (57). In addition, abatacept has also been shown to stabilize β1-integrin activation and reduce proteinuria in podocytes of patients with CD80-positive glomerular disease (58). The fusion protein targeting CD80 also holds great promise for application in autoimmune diseases.

### 2.2 Monoclonal antibody immune checkpoint inhibitors

#### 2.2.1 Application of anti-CD80 monoclonal antibodies in tumor treatment

CD80 plays a crucial role in regulating and activating both normal and malignant B cells (59, 60). CD80 is expressed on various B-cell lymphomas, including follicular, diffuse, small noncleaved, mantle cell, small lymphocytic, and other subtypes (61, 62). Galiximab (IDEC-114) is a primatized anti-CD80, immunoglobulin G1 lambda monoclonal antibody. Preclinical studies have shown that in an immunodeficient mouse model of human lymphoma xenograft, mice treated with galiximab had significantly prolonged disease-free survival (DFS) compared to untreated control mice. Similarly, mice treated with galiximab and rituximab (an anti-CD20 monoclonal antibody) had a significantly longer DFS compared to those treated with rituximab alone (63). Promising preclinical studies have quickly advanced galiximab into clinical trials. In 2005, the results of a phase I/II clinical trial of galiximab monotherapy for relapsed or refractory follicular lymphoma (FL) showed a favorable safety and efficacy profile, supporting further evaluation of galiximab as a treatment option for FL. The study also suggested that combining galiximab with other drugs may achieve better efficacy (64). Subsequently, a phase I/II preclinical trial of the combination of galiximab and rituximab in relapsed or refractory follicular lymphoma showed that galiximab could be safely combined with a standard course of rituximab (NCT00048555). This dual biologic approach offers the potential to avoid or delay chemotherapy, or to integrate with other lymphoma therapies (65). Some studies have also shown that for untreated follicular lymphoma, the extended induction regimen of galiximab-rituximab is well tolerated, and especially effective for patients with low-risk follicular lymphoma International Prognostic Index (FLIPI) score (66). In addition, anti-CD80 monoclonal antibodies have also shown good efficacy in other tumors. Some researchers have constructed mouse monoclonal antibodies against human CD80 and studied their effects on tumor growth and migration. Mouse anti-human CD80 monoclonal antibodies can specifically recognize tumor cells that naturally express human CD80 molecules, such as Burkitt’s lymphoma cells and multiple myeloma cells. It can bind CD80 on the membrane of tumors to inhibit the proliferation and migration of tumor cells and promote their apoptosis (67).

#### 2.2.2 Application of anti-CD80 monoclonal antibodies in autoimmune diseases

Monoclonal antibodies against CD80 also play an important role in autoimmune diseases. In one study, researchers constructed and characterized a monoclonal antibody (clone 4E5) against human CD80 and found that it suppressed immune responses and reduced the severity of lupus-like disease. It indicates that blocking the CD80/CD28 costimulatory signaling pathway with 4E5 is a very promising strategy to slow the progression of lupus-like diseases and other autoimmune diseases (68). Some studies have also shown that the survival time of skin grafts can be prolonged by using anti-CD80 monoclonal antibodies and cyclosporin A (69). Pathological T cell activation is associated with psoriasis progression. Relevant experimental data suggest that IDEC-114 also has promising clinical activity in patients with psoriasis (70, 71). CD80, a costimulatory molecule involved in T cell activation, may play a key role in diseases related to T cell activation.

### 2.3 Bi-or tri-specific antibodies

In addition to monoclonal antibodies, bi-specific and tri-specific antibodies associated with CD80 (CD3-CD28-CD38) are currently in preclinical testing. The CD80 ligand CD28, as an activation costimulatory signal on T cells, is also noteworthy in the use of various disease therapies. The investigators added a CD28 binding domain to the bispecific antibody to construct a trispecific antibody. Trispecific antibodies are composed of multiple structural domains, including CD38, TCR-CD3, and CD28. Compared with bispecific antibodies, trispecific antibodies can enhance the activation of T cells through TCR-CD3 and CD28 and promote the proliferation of T cells. What’s more, it can also enhance the expression of anti-apoptotic protein Bclxl in T cells, and improve the ability of T cells to kill different myeloma cell lines *in vitro* and humanized mouse models (72). The main reason for including the CD28 domain in the trispecific antibody was to mimic the second signal of T cell activation and allow better T cell activation. We can speculate that the activation of CD28 is so crucial that CD80 as its ligand may bring more possibilities in tumor treatment.

### 2.4 CAR T-cell therapy

In addition to being used in antibodies, CD28, the ligand of CD80, has also been used in cell therapy. The CD28 costimulatory signal is integrated into another type of immunotherapy called chimeric antigen receptor T-cell (CAR-T) therapy. The structure of CAR-T includes both cancer-cell antigen recognition domains and T-cell activation domains. T-cell activation domains include CD3, 4-1BB, and CD28. Second-generation CAR-T containing CD28 costimulatory molecule can be better activated when exposed to CD80 stimulation on the surface of tumor cells, which makes the second-generation CAR-T cells much stronger than the first-generation CAR-T cells, and also shows better therapeutic effects in clinical treatment. In addition, dual-target dual-signal CAR-T has also achieved better efficacy. Some researchers have found that CAR-T cells target two tumor-associated antigens at the same time, provide co-stimulation of CD28 and 4-1BB respectively, and share a CD3ζ chain, which can rapidly exert anti-tumor effects under *in vivo* stress conditions, protect tumors from re-attack, and prevent tumor escape due to low antigen density (14). This mode of “split costimulation” of the CD28 signal and 4-1BB signal makes CAR-T anti-tumor efficacy better and has been confirmed to be universal (14, 73). The activation of CD28 is so crucial that we wonder whether we can start from CD80 to better activate CD28 through CD80 to improve the efficacy of CAR-T. This provides another approach to address the suboptimal response to CAR T therapy in solid tumors.

CAR-T therapy also plays an important role in autoimmune diseases. Recently, a researcher used CRISPR-Cas9 gene editing technology to genetically engineer healthy donor-sourced CAR-T cells targeting CD19 to develop a new generation of allogeneic universal CAR-T therapies that helped three patients with autoimmune diseases achieve long-term remission (74). Autoreactive B cells play a key role in the pathogenesis of autoimmune diseases such as systemic lupus erythematosus, rheumatoid arthritis, and multiple sclerosis. Recent studies have also shown that CD19 CAR T cells targeting B cell antigen CD19 have a good effect on crescentic glomerulonephritis (75), multiple sclerosis (76), systemic lupus erythematosus (77), and autoimmune rheumatic diseases (78). In addition, several clinical trials of CAR-T therapy for autoimmune diseases are underway, indicating that the further exploration of CAR-T cells for autoimmune diseases is very promising. Therefore, enhancing the efficacy of CAR-T by CD80 stimulation of CD28 may promote the progress of CAR-T immunotherapy in autoimmune diseases.

One of the effects of CD80 on CAR-T cells is to make them better activated via CD28. We speculate that this may make CAR-T cells over-activated. On the one hand, this over-activation may lead to faster inactivation of CAR-T, which may result in poor anti-tumor efficacy. On the other hand, a large number of CAR-T cells releasing a large number of cytokines along with their activation may lead to the emergence of a cytokine storm.

### 2.5 Vaccines

#### 2.5.1 CD80-related vaccines for tumor

One reason the immune system cannot wipe out tumors is that T cells encounter tumor antigen-derived peptides on the surface of tumor cells in a tolerant but not activating environment since tumor cells do not express T-cell costimulatory molecules such as CD80. Overcoming tolerance to tumor-associated antigens remains an obstacle to cancer vaccine-based immunotherapy. One strategy to enhance antitumor immune responses is to add adjuvants to cancer vaccine regimens. The important role of CD80 in T cell activation makes it an option for tumor vaccine adjuvant.

CD80-related hematological vaccine has also shown good efficacy in preclinical studies. The CD80 gene was transfected into the K562 cell line by electroporation. It was found that the stimulation of CD80 could Induce toxic effects of natural killer cells on CD80+K562 cells and suggested that these cells may be used for further development of therapeutic tumor vaccine (79). Subsequent studies have shown that leukemia cell vaccines co-expressing CD80 and GM-CSF can be used for immunotherapy of Ph+ ALL (acute lymphoblastic leukemia, ALL) patients, which can strongly inhibit the progression of leukemia. This improves the long-term survival rate by 40-60% (80). Similarly, Acute myeloid leukemia (AML) blasts modified with IL-2/CD80 as a vaccine *in vitro* can induce peripheral blood mononuclear cells from AML patients to secrete higher levels of IFN-γand show stronger lytic activity against autologous, unmodified blasts (81). It is concluded that CD80 has a good prospect in the research of hematoma vaccine.

CD80-related vaccines have also been used in solid tumors. As early as 1997, studies showed that IL-2(+) and CD80(+) tumor vaccines could protect and prolong the survival time of a higher proportion of sarcoma-bearing mice compared with IL-2(+) tumor vaccines (82). Westermann, J et al. constructed a novel breast cancer vaccine that co-expressed MUC1 and CD80. It could elicit tumor-specific immune responses, which were closely related to B7 molecules (CD80), and suggested that the vaccine might be a good candidate for immunotherapy of MUC1-positive breast cancer (83). Preclinical studies have shown that the allogeneic tumor cell line RCC-26 exhibits natural immunogenic potential, which is enhanced by the expression of CD80 costimulatory molecules and IL-2 secretion. Subsequently, they reported the study of RCC-26/CD80/IL-2 cells in a phase 1 vaccine trial for patients with metastatic renal cell carcinoma (mRCC). The results showed that the disease was stable and the vaccine was safe, further demonstrating the feasibility of CD80 application in tumor vaccines (84, 85). Some studies have also shown that CD80-Fc can enhance T cell immune responses to a variety of tumor-associated antigens including Survivin and HPV, showing its potential as a universal adjuvant for tumor vaccines (86). Thorne, AH et al. constructed DC-PC-3 fusion vaccines with specific modifications of CD80 and GM-CSF that strongly promoted T cell proliferation and IFN-γ secretion and induced tumor-specific cytotoxic T lymphocyte responses. In addition, the CD80/GM-CSF modified fusion vaccine showed more significant anti-tumor effects and a stronger ability to stimulate immune responses *in vitro* than the vaccine without specific modification (87).

These studies have shown that CD80-related vaccines play a crucial role in the activity of T cells, and play an important role in solid tumors or hematomas, which may provide a new development direction for tumor immunotherapy.

#### 2.5.2 CD80-related vaccines for autoimmune diseases

Hiv-specific CD8+ T cells are deficient in chronic HIV infection. Studies have shown that co-stimulation of CD80 enhances the acquisition of antigen-specific amplification and effector function of HIV-specific memory CD8+ T cells, which represents a promising therapeutic HIV vaccination combination (88). In other autoimmune diseases, there is no relevant research on CD80-related vaccines. CD80 may provide a new direction for the prevention and treatment of other autoimmune diseases.

## 3 Conclusion and outlook

**Figure 1:**
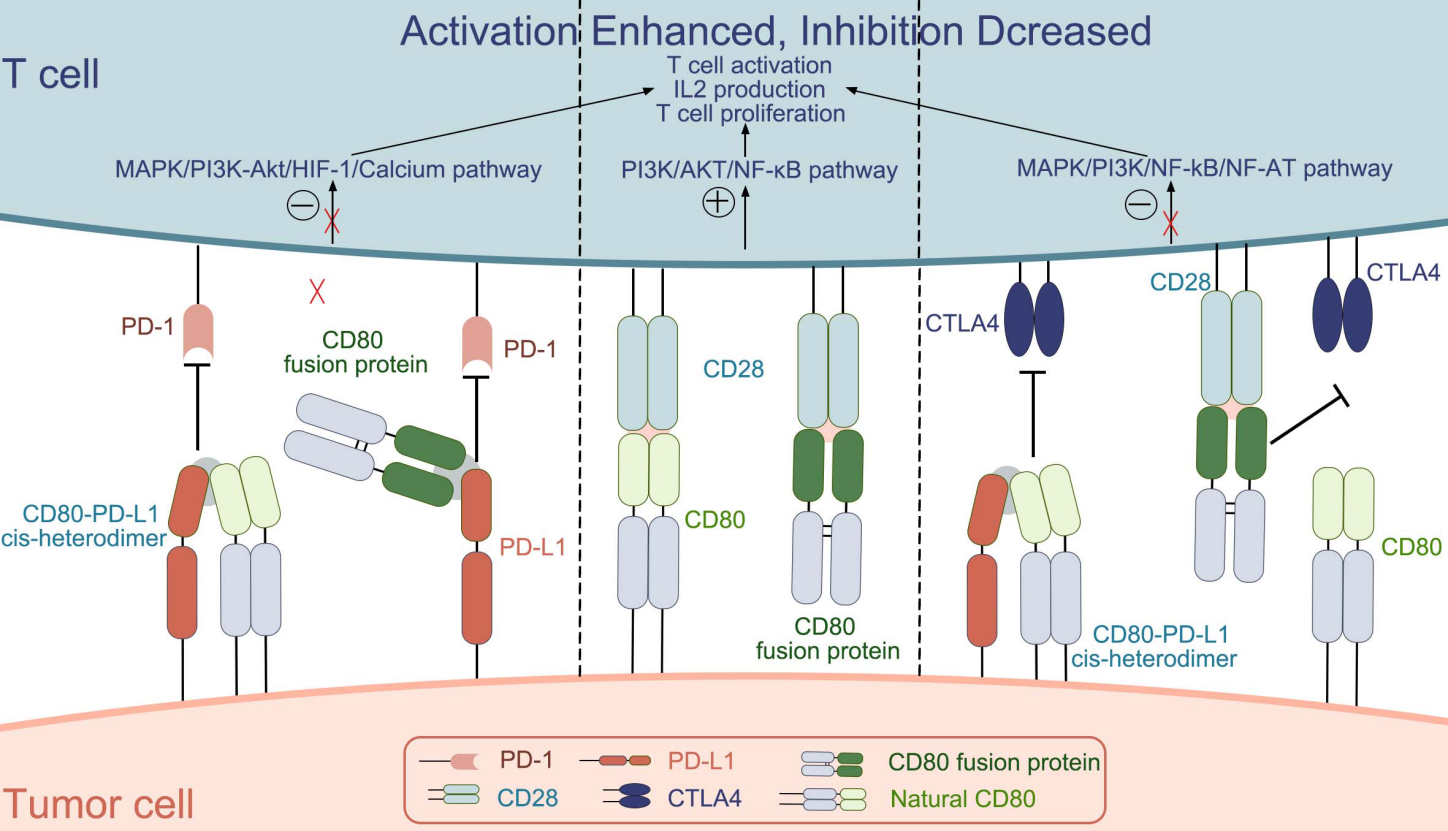
The interactions between CD80 (natural CD80 and CD80 fusion protein) and the molecules CD28, CTLA4, and PD-L1.[.

The CD80 protein is expressed on the surface of antigen-presenting cells as well as within the innate immune system. Its role is to establish a biologically optimal dynamic balance between the activation of the immune system and its suppression or self-tolerance. The interaction between B7-1 and its receptors CD28, CTLA4, and PD-L1 can stimulate or suppress immunity or regulate immune homeostasis (**Figure 1**). Based on the study of CD80 physiology and pathology, CD80 or its related ligands for drug development is a good direction for immunotherapy.

CD80 plays an important role in T cell activation. On the one hand, as a ligand for the costimulatory molecule CD28 on the surface of T cells, CD80 can enable better activation of T cells. On the other hand, it binds to PD-L1 on the surface of tumor cells to prevent PD-L1 from binding to PD-1 on T cells and reduce T cell inhibition. Thus, CD80 plays an important role in tumor therapy. The CD80 fusion protein, bi/tri-specific antibody, CAR-T, etc. all play a role in the development of tumor immunotherapy. Although clinical trials of CD80 fusion protein have demonstrated its efficacy, its safety needs to be further investigated. CD80-related bi/tri-specific antibodies are still in preclinical research and need to be further improved. Although CAR-T therapy has a good effect on hematological tumors, its safety and efficacy in solid tumors need to be further improved. Therefore, the combination of CD80 and CAR-T therapy may have a better effect.

In addition to the physiological role of CD80, the pathological role of CD80 was also investigated. The expression of CD80 on the surface of a variety of tumor cells makes it an alternative target for tumors. The development of new strategies for tumor immunotherapy targeting CD80 has good application prospects. Monoclonal antibodies targeting CD80 have entered clinical trials for the treatment of lymphoma, but the safety of CD80 as a target needs to be further investigated. In addition, abnormal CD80 is also closely related to autoimmune diseases, which makes targeting abnormal CD80 molecules in autoimmune diseases an option for the treatment of autoimmune diseases. Its preclinical data showed promise, but its safety needs to be further explored. In recent years, cancer vaccines have been under investigation. The expression of CD80 on the tumor surface and its important role in T cell activation also make it a good choice for tumor vaccine adjuvant.

Natural CD80 exists as a dimer on the cell membrane, and the main targets that can be bound are CD28, CTLA-4 and PD-L1.CD80 binds differently to the three targets, with KD values of 4 μM, 1.7 μM, and 0.2 μM; for binding to CD28, PD-L1, and CTLA4, respectively, with the strongest ability to bind CTLA-4 (25). The interaction between CD80 and CD28, induces the activation of nuclear factor-κB (NF-κB), mitogen-activated protein kinase (MAPK), and the calcium–calcineurin pathway, thereby playing an important role in manipulating the immune system (25). In addition, some researchers have also investigated the molecular mechanism of CD80 in the immune process. They found that CD80 activation was closely associated with the PI3K → AKT → NF-κB pathway and NOTCH signaling (89). Few studies have reported the molecular mechanisms behind CD80 interactions with PD-L1 and CTLA-4, this will be a direction we will explore further in the future.

There are still challenges in developing CD80-related drugs. Firstly, CD80-associated drugs are usually dose-limited in clinical trials, suggesting that appropriate concentrations of CD80-associated drugs are critical to the safety of therapy. CD80-related drugs have been reported in clinical studies to cause immune-mediated myocarditis or infections that can lead to patient death (NCT04186637). This may be due to the fact that CD80-related immunotherapy drugs bind to the CD28 molecule causing T cells to overactivate and secrete large amounts of cytokines thereby generating a cytokine storm that can lead to patient death. On the other hand, we speculate that over-activation of T cells will make them inactive earlier, which may lead to poor anti-tumor efficacy. Secondly, there is a limited effect of CD80-related drugs alone in the treatment of tumors for the reason that it does not have sufficient affinity for the ligand or has a short survival time *in vivo*. So, increasing the affinity of CD80 as well as prolonging its half-life is also critical for therapeutic efficacy. It is now clear that further improvements in rates and durability of responses may require combining CD80 with other therapies that stimulate a proinflammatory immune response within the tumor microenvironment. Finally, cells of different tumor types evade antitumor immunity via different expression of CD80. For example, the high expression of CD80 on the cell surface of tumor cells significantly improved the overall survival of patients, while in other tumor cells, CD80 overexpression was an indicator of poor prognosis. Therefore, we need to explore the relationship between CD80 expression and prognosis in different tumors.

Addressing the gaps needs to be followed up with further research. For example, in the case of CD80 fusion protein, we can change its affinity, concentration, half-life, and so on, so as to make it more effective, gentle, and sustained in killing tumor cells while reducing the side effects. Using CD80 as a target, or as a T-cell efficacy enhancer is also a solution to the CAR-T therapy in the dilemma of treating solid tumors.
