# Corrigendum: Research progress of CD80 in the development of immunotherapy drugs

**DOI:** 10.3389/fimmu.2024.1536312

**Published:** 2024-12-05

**Authors:** Lanying Li, Lei Yang, DePeng Jiang

**Affiliations:** Department of Respiratory Medicine, The Second Affiliated Hospital of Chongqing Medical University, Chongqing, China

**Keywords:** CD80, immunotherapy drugs, T cells, tumor, autoimmune diseases

In the published article, there was an error in the **Funding** statement. “The Kuanren Talents Program of the Second Affiliated Hospital of Chongqing Medical University”. The correct Funding statement appears below.

“The author(s) declare that financial support was received for the research, authorship, and/or publication of this article. This study was supported by The First batch of key Disciplines On Public Health in Chongqing”.

The authors apologize for this error and state that this does not change the scientific conclusions of the article in any way. The original article has been updated.

