# Corrigendum: Research progress of CD80 in the development of immunotherapy drugs

**DOI:** 10.3389/fimmu.2024.1538349

**Published:** 2024-12-18

**Authors:** Lanying Li, Lei Yang, DePeng Jiang

**Affiliations:** Department of Respiratory Medicine, The Second Affiliated Hospital of Chongqing Medical University, Chongqing, China

**Keywords:** CD80, immunotherapy drugs, T cells, tumor, autoimmune diseases

In the published article, there was an error in the affiliations. Instead of “Institute of Cancer, Xinqiao Hospital, Army Medical University, Chongqing, China”, all authors should only be affiliated to “Department of Respiratory Medicine, The Second Affiliated Hospital of Chongqing Medical University, Chongqing, China”.

In the published article, there was also an error to the Funding section and “Application of single cell biomolecular analysis in the pathogenesis of lung cancer (cstc2022yejh-bgzxm0051)” was accidently omitted.

The corrected funding appears below:

The author(s) declare that financial support was received for the research, authorship, and/or publication of this article. This study was supported by The First batch of key Disciplines On Public Health in Chongqing and the Application of single cell biomolecular analysis in the pathogenesis of lung cancer (cstc2022yejh-bgzxm0051).

The authors apologize for these errors and state that these do not change the scientific conclusions of the article in any way. The original article has been updated.

